# Male involvement during pregnancy and childbirth: men’s perceptions, practices and experiences during the care for women who developed childbirth complications in Mulago Hospital, Uganda

**DOI:** 10.1186/1471-2393-14-54

**Published:** 2014-01-31

**Authors:** Dan K Kaye, Othman Kakaire, Annettee Nakimuli, Michael O Osinde, Scovia N Mbalinda, Nelson Kakande

**Affiliations:** 1 Department of Obstetrics and Gynecology, School of Medicine, College of Health Sciences, Makerere University, P.O. Box 7072 Kampala, Uganda; 2 Department of Obstetrics and Gynecology, Jinja Regional Hospital, Jinja, Uganda; 3 Department of Nursing, School of Health Sciences, College of Health Sciences, Makerere University, P.O. Box 7072 Kampala, Uganda; 4 Clinical, Operations and Health Services Research Program, Joint Clinical Research Centre, P. O. Box 10005 Kampala, Uganda

## Abstract

**Background:**

Development of appropriate interventions to increase male involvement in pregnancy and childbirth is vital to strategies for improving health outcomes of women with obstetric complications. The objective was to gain a deeper understanding of their experiences of male involvement in their partners’ healthcare during pregnancy and childbirth. The findings might inform interventions for increasing men’s involvement in reproductive health issues.

**Methods:**

We conducted 16 in-depth interviews with men who came to the hospital to attend to their spouses/partners admitted to Mulago National Referral Hospital. All the spouses/partners had developed severe obstetric complications and were admitted in the high dependency unit. We sought to obtain detailed descriptions of men’s experiences, their perception of an ideal “father” and the challenges in achieving this ideal status. We also assessed perceived strategies for increasing male participation in their partners’ healthcare during pregnancy and childbirth. Data was analyzed by content analysis.

**Results:**

The identified themes were: Men have different descriptions of their relationships; responsibility was an obligation; ideal fathers provide support to mothers during childbirth; the health system limits male involvement in childbirth; men have no clear roles during childbirth, and exclusion and alienation in the hospital environment. The men described qualities of the ideal father as one who was available, easily reached, accessible and considerate. Most men were willing to learn about their expected roles during childbirth and were eager to support their partners/wives/spouses during this time. However, they identified personal, relationship, family and community factors as barriers to their involvement. They found the health system unwelcoming, intimidating and unsupportive. Suggestions to improve men’s involvement include creating more awareness for fathers, male-targeted antenatal education and support, and changing provider attitudes.

**Conclusions:**

This study generates information on perceived roles, expectations, experiences and challenges faced by men who wish to be involved in maternal health issues, particularly during pregnancy and childbirth. There is discord between the policy and practice on male involvement in pregnancy and childbirth. Health system factors that are critical to promoting male involvement in women’s health issues during pregnancy and childbirth need to be addressed.

## Background

Male involvement in pregnancy and childbirth influences pregnancy outcomes [1-6]. It reduces negative maternal health behaviors [2-7], risk of preterm birth, low birth weight, fetal growth restriction and infant mortality [2-7]. There is epidemiological and physiological evidence [7,8] that male involvement reduces maternal stress (by emotional, logistical and financial support) [1,4], increases uptake of prenatal care [9], leads to cessation of risk behaviors (such as smoking) [9,10], and ensures men’s involvement in their future parental roles from an early stage [11-14].

While there are reports of negative experiences (such as being ignored or marginalized [11]), there are reports of positive experiences (such as receiving timely attention and adequate support from healthcare providers [12-16]. Negative experiences may lead to difficulty in men’s and women’s adjustment to pregnancy, childbirth and parenthood (consequently limiting the support men provide to their partners in the postpartum period) [17-21]. Uganda’s Ministry of Health has a policy that supports male involvement in reproductive health. The ways in which the policy is implemented might discourage men from playing active roles [22]. Despite the importance attached to men’s involvement in Uganda, there is limited research on male involvement during pregnancy and childbirth in Uganda, particularly from the men’s perspective, which hampers development of contextualized appropriate interventions. The objective was to gain a deeper understanding of male involvement during pregnancy and childbirth by exploring men’s perceptions, experiences and practices.

## Methods

### Study setting

The study was conducted in from April 1-June 15, 2013 in Mulago hospital, Uganda. Mulago is the national referral hospital and the teaching hospital for Makerere University. The hospital has over 1500 beds, of which over 400 are maternity beds. However, due to the large number of hospital admissions, there congestion on the wards and floor cases are common. Mothers with severe acute obstetric complications are admitted in the high-dependency unit, a six–bed semi-autonomous unit. Here they are offered closer observations and more frequent reviews by a team of specialist obstetricians, residents, intern doctors and midwives. This unit often allows female attendants to stay with the admitted patients. Males are only allowed short visits at visiting hours (early in the morning, over the lunch hours and in the evenings).

#### The study participants

The study participants were 16 men who came to the hospital to visit their spouses/partners who had been admitted with severe complications of pregnancy or childbirth to the high-dependency unit. None of these men was a full-time patient attendant. Participants were approached by a research assistant (a midwife on the emergency unit) and requested to participate in the study. None of the men approached declined to participate. Having agreed to participate, they were given assurance that the information given would be confidential, that their views would be anonymous, and that declining participation would not in any way prejudice the care their patients were to receive at the hospital. They were requested for permission to record the proceedings, after which they signed an informed consent. The interview guide was piloted on four men who had patients in the general maternity ward (whose spouses had an uncomplicated deliveries) to assess acceptability, feasibility and content of the questions.

#### Data collection

Participants were selected purposefully by maximum variation sampling to represent a variety of age groups, education level and socio-economic status. All the interviews took place on the day participants were approached, and were conducted in one of the private offices on the ward by one of the authors (DKK). We employed a phenomenological approach to assess participants’ perceptions, experiences and practices, specifically using Schutz’s social phenomenology [23]. This is a descriptive and interpretive theory of social action that explores subjective experience of individuals and meanings they attach to the experiences. Schutz’s theory emphasizes the spatial and temporal aspects of experience and social relationships. Social phenomenology takes the view that people living in the world of daily life are able to ascribe meaning to a situation and then make judgments. We explored the participants’ description of the relationship with the patient, their roles and responsibilities (during pregnancy and childbirth), and the specific activities they undertake (in relation to the spouse’s healthcare). We further explored perceived specific benefits and barriers to men’s involvement in their spouse’s healthcare, as well as perceived as strategies to increase male involvement during pregnancy and childbirth. Interviews lasted for 30-50 minutes and were conducted in English and Luganda, a widely spoken Bantu language. The proceedings were tape recorded. At the end of each interview, the key points were summarized to the participant in order to verify the data. Hand-written notes were taken during the interviews.

#### Data analysis

Data was analysed using deductive content analysis [24]. The transcripts were read to identify patterns of words, phrases or statements across dataset that were important description of the essence of the phenomenon. Such constellation of words or statements relate to the same central meaning. These constituted the meaning units and contained aspects related to each other through both their content and context. Codes were assigned to these meaning units. Similar or related codes were aggregated later into categories and themes through a method of abstraction [25]. The agreement on which codes and categories were generated was by consensus. The codes were aggregated according to the identified meaningful patterns into categories that were exhaustive and mutually exclusive. A theme was identified as a consistent pattern found in the information that described or interpreted aspects of the phenomenon [24,25]. Handwritten notes recorded the body language, the approach to responses (such as eagerness or hesitancy) and the participants’ mood during the conversations, so that the responses could be interpreted in the context in which they were made. A deductive approach is useful if the aim is to test an earlier theory in a different situation. Easy Text (EZ) software was used for data retrieval during analysis.

#### Ethical considerations

This research was part of a post-doctoral research project of the first author (DKK) entitled *Evaluation and surveillance of the impact of maternal and neonatal near-miss morbidity on the health of mothers and infants in Jinja and Mulago hospital.* Ethical approval to conduct the study was obtained from the Ethics and research committees of Mulago hospital, the School of Medicine, Makerere University College of Health Sciences and Uganda National Council for Science and Technology. All participants gave written consent to be interviewed and for proceedings to be recorded, and were given assurances that their participation was voluntary.

## Results

In this study, an ideal father is described by participants as being accessible (present, available, a team player) and responsible (is concerned, maintains connection with the woman carrying the child regardless of the partnership status, and is a caregiver, provider or protector). The ideal father expects and wants to be engaged (cares about the pregnancy and the coming child, wants to learn more about the process, wants to be involved and wants to be supported by the health system to accomplish his roles). Men are willing and eager to support their partners/wives/spouses at this time, but are hindered by health system factors mainly, in addition to personal, relationship, family and community constraints. We identified the following themes: Men have different descriptions of their relationships; responsibility was an obligation; ideal fathers provide support to mothers during childbirth; the health system limits male involvement in childbirth; men have no clear roles during childbirth, and exclusion and alienation in the hospital environment.

### Men have different descriptions of their relationships

Participants expressed different descriptions of their relationships with the spouses. They described themselves as “husbands”, “partners” or “spouses” of the women. Others described themselves as the “biological father of the unborn child”, “father of her children”, “father of the children” or a “lover”. Several participants reported that on many occasions, the “father” or “male partner” was not the spouse/husband, such that (this and what they perceived as the nature of the relationship) might affect the degree of male partner involvement in the spouse’ healthcare. The father was perceived to provide care for the partner and was willing to support her during that period, irrespective of whether he was the biological father. This is exemplified by one man (who did not know the second name of the “partner” with whom he had stayed for only six months, but was coming to visit her during childbirth):

“The “ideal” situation is that the “love partner” and the “biological father” are the same person. However, this is not always the case. I found her with a pregnancy. I stay with her. Now that I am involved, I will provide care. The relationship is “good”. I will support her even if I am not responsible (not the biological father of the child)”.

### Responsibility is an obligation

There general consensus was that should a man become involved with a pregnant woman, he would also accept “partnership” in the pregnancy and (as her “partner”). This was perceived as a man’s obligation. If the man was responsible for the pregnancy, he was required to demonstrate that responsibility by supporting and caring for the woman during pregnancy and childbirth. This obligation extended to any man involved in a relationship with a pregnant woman, even if he was not the “biological father” of the unborn child. Such a man would take on the responsibility to support the woman throughout the (pregnancy and childbirth) process, particularly when she gets complications. Therefore, expectations about a “love partner” were similar to those about the “biological father”. This is exemplified by one young respondent, aged 20 years, describing himself as a ‘love partner’:

“I don’t see any difference. I care as much as I can. I am “part” of the child’s life. Whether I am responsible or not, I am assuming I am and take care of her as (and I assume) these roles.

The participants appeared concerned about the severe complications their partners had developed. They expressed anxiety about their wives’ illness, were eager to support their partners/wives/spouses at this time, and were eager to learn about the patients’ condition. They however identified personal, relationship, family and community commitments which acted as barriers to their full involvement in the patients’ healthcare. They insisted that being a “father” was more about caring than about fulfilling a duty. This is exemplified by one man who described himself as a ‘spouse”:

‘I am conscious of my role and what she expects from me. However, I have to continue with work. I have to send what they need. I am always in (phone) contact with her and what is going on. When I return home from work at the end of the day and on weekends, I attend to her. I pray it (the sickness) does not interfere and disrupt the routine. My ultimate goal is to support my wife’.

### Ideal fathers are involved and provide support during childbirth

Requested to give characteristics of an ideal father in the context of pregnancy and childbirth, participants described in detail the activities, attitudes and behaviors that demonstrate ideal male involvement. This was in response to a specific question*: What is an ideal father and what is expected of an ideal father?* The ideal father was described as someone who cared, provided emotional and financial support, and tried as much as possible to find out what was wrong with the patient. In case of complications, he sought solutions. He was described as “*considerate*”, “*present*”, “*able*” and “*accessible”*. Such a person would not necessarily be physically available but should get updates on their spouses’ situation. That is, they should keep abreast of the condition of their spouses at all times. One participant’s response sums this view:

“He is not necessarily available….he should always be aware of what is going on”

Therefore, even when they were not present at the spouses’ side physically, they kept abreast of the developments and were not absent deliberately. They receive regular communication about the patients’ (spouse’s) condition from attendants who stay with the patient all the time, these attendants are often the man’s relatives. This situation is exemplified by one respondent, a teacher, who described himself as a “*partner”* to the admitted patient, and “*part of the healthcare team*”. He believed an ideal father was a man who was there for their spouse all the time:

“…..goes (with the spouse) to the (medical) appointments when he can; ….. is accessible and available. He communicates. He is concerned…..He is in contact with the health workers (on the ward where the patient is admitted). He doesn’t desert….should be on standby to provide help. …may send a sister or mother to stay with the patient.

The ideal father’s roles were described as “*active*” during antenatal care and childbirth. Other roles were “*companion*”, “*supporter*” and “*provider*”. Most participants reported that they “*fall short of the ideal father*”. However, they believed that they made attempts to fulfill their responsibility, in spite of constraints such as job obligations, family obligations and busy schedules. Their expression of this was that they *“try”, “endeavor”* and are *“eager to learn”.*

However, this participant acknowledges that it was impossible for men to fulfill this responsibility due to other personal, family or social commitments and responsibilities:

“Many men do not know what is going on…but I am eager to learn more about the process and …always (try to) provide what is required. It is not always easy,…drugs and treatment are costly…I am willing to sacrifice…At home I help where I can., I ensure she eats well and gets enough rest….I am part of a team”.

The issue of maturity was considered critical to the ideal father role, as most participants thought that younger men were ill-prepared to handle the financial, social and emotional responsibilities as well as the roles of fatherhood. The emotional bond was believed to predict male involvement, such that men who were not committed to the relationship, irrespective of the physical age, could not effectively fulfill their responsibilities as fathers at this critical period. However, in spite of their age, men of a young age fulfilled their responsibilities as long as the emotional bond was strong. This emotional bond, however, could be affected by some of the physical, emotional or psychological changes in the women during pregnancy. An ‘ideal father’ was a manifestation of maturity and commitment to the relationship. This is exemplified by an elderly father:

“The father should be present to support, to understand, to provide financial support, to be patient, and to have ‘sympathy’. This does not happen in young people or those who are not ready. Love is very important. The relationship to the mother has a lot to do. But it is not easy to understand women at that time (pregnancy and childbirth)”.

### Failure of the health system to meet needs of men who want to be involved

The participants were of the view that it was apparent that they were expected to participate and be actively involved in the healthcare of their spouses. They indicated that messages on radio and television, articles in newspapers and materials posted on information charts in several areas of the hospital supported male involvement in reproductive health. Therefore, the contemporary societal expectations encouraged male participation in childbirth. However, the cultural and societal values were unclear regarding this expectation. The evidence for this view was the absence of clear roles for men during pregnancy or childbirth. The men often felt distanced from their partners during pregnancy, childbirth and postpartum period. As they approached birth, some mothers moved from the men’s home to stay with the women’s relatives who would support them during the labour process. If the men accompanied their partners to the antenatal clinics, they generally stayed outside the clinic, as there was no space or seat for them. The men reported that on several occasions, they were not given any particular attention. Additionally, several men thought that a lot of time was wasted in the antenatal clinic, yet they needed to spend as little time as possible in order to go for work. A participant described this situation as follows:

“When you go with her, you spend the whole day. Many times you don’t know why that time was wasted. When she feels unwell during pregnancy, she goes to her parents’ home. That is what everyone expects. She thinks she can get more help there. Even in the previous deliveries, she and her parents expected her to go there”.

Although Mulago hospital’s hospital policy encourages fathers to support their wives during pregnancy and childbirth, the presence of fathers in delivery rooms was limited because of congestion and the need to maintain privacy. This tended to cause confusion to many men, in that there was variance between the policy and the practice. Many men felt helpless, with nowhere to get information about their patients. They were often locked out of the wards. They expressed their feelings about this as being left *“anxious*”, *“helpless”* and *“useless”.* They felt alienated by the general term of *“visitors”*, and often felt like “*uninvited guests”.* This is exemplified the despair described by one participant. This man had left his wife who had been referred to the hospital with poor progress of labor and needed an emergency operation in the morning. He found that the wife had not had the operation more than eight hours later:

“I am just confused. My wife came last night. She was told she will be operated. It is now 8 hours ago. I can’t go to see her. They said that men are not allowed (in the labor ward). I want to see her. They said she should not eat, that she is going for operation. Up to now they have refused to let me enter (the ward). Nobody has talked to me. I was not prepared for this. There is no information. Her phone is off…She does not even know (that) I am around. It is very frustrating… I don’t know what is going on….I don’t know what to do”.

### Unclear roles in childbirth are a deterrent to male involvement

The perceived major deterrents to men’s involvement during childbirth were personal factors (such as unhealthy couple relationship) or unclear roles (not knowing what the healthcare system expected of them, lack of information on what to expect, not knowing their role or not wanting the new responsibilities). Many men felt they also need not only information but also support during childbirth, so that they can effectively support their partners. In addition, many men reported wanting to be present or to participate in their partners’ labor and delivery, and to be actively involved in the decision-making. One young man had this to say, exemplifying this challenge:

“I do not know what is happening. The previous delivery was normal, thought she (the wife) was operated on the first pregnancy. We were sent here because she had poor progress. She called me (on phone) that she would be taken for operation. It is now 5 hours and I do not know what is going on. ‘Doctors’ do not involve us in the decision (healthcare decisions). It is not good to ask questions”.

#### Exclusion and alienation

Regarding their experiences of the hospital environment and the services provided, in relation to male involvement, all the participants expressed sentiments that they were excluded from what was going on, particularly from the decision-making process regarding the care given to their patients. The hospital environment, the behavior and language of the healthcare providers appeared to increase the participants’ feeling of alienation. One respondent, who escorted the spouse to four antenatal visits, had this complaint:

*“They (health workers) should be more humane, more supportive. There (in the antenatal clinic) they were welcoming. We need information (now that the wife was in labor) but can’t get it. In the antenatal classes, they never told us we will go through this. You wonder what your role is. You get so stressed. You don’t know what is happening. It is frustrating*”.

There few attempts to give information to men about their partners’ condition. Where this was done, the information was perceived as inadequate. One apparently aggrieved participant, whose wife had severe preeclampsia on the fourth pregnancy, was very bitter. He described himself as the husband and had this to say:

“I was only told that I have to sign the form (consent form) because my wife had ‘pressure’ (high blood pressure). She is very sick so I don’t think she made any decisions (was involved in the decision-making). I am not involved in any decisions made regarding her care…. They only told me the baby is dead but she (the spouse) still needs (to undergo an) the operation). When you ask when (the operation will be done), you get no answer”.

However, some participants felt deep fear about witnessing something going wrong, and were not so eager to be present during delivery. They believed that doctors knew what to do and would do what is best for the patients. The major sentiment was that while ‘doctors’ knew what they were doing (had the expertise), they should not make men passive recipients of care. The doctors should at least inform the men about major decisions taken regarding their partners healthcare*.* The participants further suggested that there should be programs which provide information about this period (childbirth) to men, or which provide counseling and support.

However, some participants believed that they have limited role during childbirth, beyond financial and emotional support. Participants were unanimous that there needed to be education for men to increase their knowledge of pregnancy and childbirth and what transpires. They also wanted full explanation on the policy which encourages male involvement and what it expects of men once they escort their spouses to the hospitals during childbirth. Participants also emphasized that health care providers should appreciate and support the involvement of men in maternal health issues during antenatal care and childbirth. Currently, this was not seen to be happening, and where attempts were made, they were viewed as ‘unsatisfactory’. Finally, participants recognized the importance of providing support and training in customer care and communication to health workers, so as to improve their relationship with secondary clients, who are partners of women during pregnancy and childbirth.

## Discussion

Men’s involvement during pregnancy and childbirth plays a vital role in the safety of their female partners’ pregnancy and childbirth, by ensuring access to care and provision of emotional and financial support [26,27] and guarantying women’s access to reproductive health services in general [28-31]. However, in agreement with prior research [32], personal, family, societal and health system factors limit male involvement. Three main qualities (access, engagement and responsibility) [32], in agreement with Lamb’s theory [33], may explain male involvement. Lamb’s theory [33] posits that the three components (engagement, accessibility and responsibility) explain the degree of fathers’ involvement with the child and its mother. The quality (emotional connection) of the fathers’ relationship with the pregnant woman on the fathers’ expectations, experiences and practices. Childbirth is the time when men are most receptive to getting involved with their families [34], which makes male involvement critical for healthy pregnancy outcomes, infant survival and ideal child development [35-40].

The study shows that despite existence of a supportive policy for male involvement, men experience stressful situations in their attempts to be involved during pregnancy and childbirth. There is dissonance between societal expectations and men’s experiences, as well as dissonance between the policy for male involvement and the practice in the health system. The men realize that the health system’s policy (that advocates for male involvement), and the contemporary societal expectations (that men should be as involved as possible in pregnancy, birth and childrearing) contrast sharply with the reality. The unwelcoming hospital environment is characterized by lack of privacy, absence of facilities in which men would be comfortable, apparent neglect by healthcare providers, lack of communication and near-total exclusion of men (who are willing to be involved) from healthcare issues of their spouses at this critical time. As in prior research [41,42], attempts at being involved might predispose men to psychological or mental scarring. This might arise from being alienated, ignored or mistreated by healthcare providers in an unsupportive hospital environment [42-45]. Regarding the suggestions to improve male involvement, our findings are in agreement with research from Malawi, Syria, Tanzania and Nigeria [20,21,26,43-46] and a review study [47] that providing motivational information, ensuring positive healthcare provider attitudes, and providing educational support and a conducive environment to men are potential interventions to increase male involvement in pregnancy and childbirth.

### Strengths and limitations

As limitations, this was a hospital-based study, and participants were restricted to men who came to the hospital, most of whom were from urban or peri-urban areas. This was a highly selected population whose views are not representative of all men or of men who do not accompany their wives during pregnancy or childbirth. Since these were spouses of women with severe life-threatening pregnancy complications, their views might differ from those of men whose wives had normal deliveries or had less severe pregnancy or childbirth complications. Likewise, views of men from rural areas might be different from those of men in urban areas. However, our study generates information on the roles, expectations, experiences and challenges of men who wish to be more involved in maternal health issues, particularly during pregnancy and childbirth. It also identifies the critical need to identify innovative ways of operationalizing the policy of male involvement in pregnancy and childbirth, with suggestions on how and what interventions could be implemented to increase male involvement.

## Conclusion

Mulago hospital should identify innovative ways of operationalizing the policy of male involvement in pregnancy and childbirth in order to effectively engage men who are keen to be involved in healthcare of their partners. This might involve health education of men who escort their partners to antenatal clinics, on expected roles during pregnancy and childbirth. The hospital should train health care providers in customer care, and needs to identify waiting rooms in which male are welcomed, provided with information on their spouses and given health education on expected roles. Health workers need to improve on customer care, particularly regarding communication of the patients’ condition or healthcare to patient attendants. The Uganda Ministry of health needs to offer health education to all men on specific roles in pregnancy and childbirth, and the importance of this role to positive pregnancy outcomes.

## Competing interests

The authors declare that they have no competing interests.

## Authors’ contributions

DKK conceptualized the study the post-doctoral research project and the study of which it is part. OK, MOO, and NK advised on the design. DKK collected the data, led the analysis, and wrote the text of the paper. All the co-authors gave advice on the data analysis, presentation of the results, reviewed and edited the text and approved the final manuscript.

## Pre-publication history

The pre-publication history for this paper can be accessed here:

http://www.biomedcentral.com/1471-2393/14/54/prepub
